# Resolution of a fungal mycotic aneurysm after a contaminated steroid injection: a case report

**DOI:** 10.1186/1756-0500-7-327

**Published:** 2014-05-31

**Authors:** George Nelson, Olga Fermo, Kiran Thakur, Elizabeth Felton, Jee Bang, Lucy Wilson, Susan Rhee, Rafael Llinas, Kristine Johnson, David Sullivan

**Affiliations:** 1 Division of Infectious Diseases, Johns Hopkins University School of Medicine, Baltimore, Maryland, USA; 2 Department of Neurology, Johns Hopkins University School of Medicine, Baltimore, Maryland, USA; 3 Maryland Department of Health and Mental Hygiene, Baltimore, Maryland, USA; 4 Johns Hopkins Bloomberg School of Public Health, Baltimore, Maryland, USA; 5 W. Harry Feinstone Department of Microbiology and Immunology, Room E5628, The Johns Hopkins Bloomberg School of Public Health, 615 North Wolfe St, Baltimore, Maryland 21205, USA

**Keywords:** Fungal polysaccharides, (1,3) beta-D-glucan, Mycoses, Infected aneurysm, Voriconazole

## Abstract

**Background:**

In the past ten years there have been three separate outbreaks of fungal contaminated steroid injections from compounding pharmacies. The 2012 outbreak of central nervous system fungal infections associated with contaminated methylprednisolone produced by a United States compounding pharmacy has led to 750 infections (151 with meningitis and paraspinal infections and 325 cases with paraspinal infections without meningitis) and 64 deaths as of October 23, 2013. *Exserohilum rostratum* has been the predominant pathogen identified by culture, polymerase chain reaction or antibody tests. According to previous reports, cerebral involvement with phaeohyphomycosis has a high risk of morbidity and mortality.

**Case presentation:**

We report a 41 year-old Caucasian woman who received a lumbar methylprednisolone injection from a contaminated lot in August 2012. She was diagnosed with fungal meningitis by cerebrospinal fluid pleocytosis and positive (1, 3) beta-D-glucan after cultures and polymerase chain reaction were negative. Two weeks after onset of therapy, she developed a 4.1 mm superior cerebellar artery mycotic aneurysm associated with new stroke symptoms, which resolved with thirty-two weeks of antifungal treatment.

**Conclusions:**

This is the rare case report of successful medical management of a cerebral mycotic aneurysm with stroke symptoms related to a presumed phaeohyphomycosis in an immunocompetent individual. Further studies are needed to determine the utility of cerebrospinal fluid (1, 3) beta-D-glucan in diagnosing and monitoring patients with meningitis thought to be related to fungal infection.

## Background

In the past ten years there have been three separate outbreaks of fungal contaminated steroid injections from compounding pharmacies in the United States [1-3]. The 2012 outbreak of central nervous system (CNS) fungal infections associated with contaminated methylprednisolone produced by a compounding pharmacy has led to 751 infections (233 with meningitis alone, 151 with meningitis and paraspinal infections and 325 cases with paraspinal infections without meningitis) and 64 deaths as of October 23, 2013 [2,4,5]. *Exserohilum rostratum* has been the predominant pathogen identified by culture, polymerase chain reaction (PCR) or antibody tests with 85% (153/180) of the identified isolates [6,7].

According to previous reports, cerebral involvement with phaeohyphomycosis has a high risk of morbidity and mortality [8,9]. Here we report the medical management and resolution of a fungal meningitis case associated with development of a mycotic aneurysm.

## Case presentation

A 41 year-old Caucasian woman with a history of Ehlers-Danlos type III syndrome, migraine headaches, and sciatica received a lumber 4/lumbar 5 translaminar epidural steroid injection from a contaminated compounded steroid lot (06292012@26) on August 31, 2012 [10]. Seventeen days after her injection, she developed headaches, nausea and vomiting and was diagnosed with sinusitis and migraines. Thirty-seven days after injection she received a notification letter of exposure from the compounding facility and presented to her local emergency department. She underwent a lumbar puncture (LP) showing pleocytosis (Table 1). Given these results and otherwise negative workup for alternative etiologies, and in the context of the ongoing contaminated steroid outbreak, she was treated with liposomal amphotericin B (5 mg/kg daily) and voriconazole (5 mg/kg IV q12h) for presumptive fungal meningitis. Repeat lumbar puncture (LP) performed one week after initiation of therapy showed no increase in pleocytosis (Table 1). She was discharged from the outside facility three days later on oral voriconazole, though medication initiation was delayed due to medication supply issues.

**Table 1:** Serial cerebrospinal fluid analysis

**Date**	**OP**	**Total WBC (x 10**^**6**^**/L)**	**N%**	**L%**	**M%**	**Glu mg/dL**	**Prot mg/dL**	**ESR/CRP**	**BDG pg/mL**	**FCx PCR**	**Cr**
Oct 8	ND	353	38	48	14	43	85		>500	Neg	
Oct 15	ND	225	67	25	8	43	79		>500	Neg	
Oct 21	17	240	69	27	4	44	138		>500	Neg	1.1
Nov 9	ND	140	41	38	21	43	180		>500	Neg	1.3
Dec 10								48/.6			1.7
Jan 21								42/.2			1.5
Jan 26	ND	8	25	0	75	54	117				
Feb 4								18/<1			1.1
Jun 19								11/<1			1

Table Footnotes: OP-opening pressure, WBC-white blood cells, N-neutrophils, L-lymphocytes, M-monocytes, ESR- erythrocyte sedimentation rate, CRP- C-reactive protein, FCx-Fungal cultures, Cr-creatinine, ND- not done.

**Figure 1:**
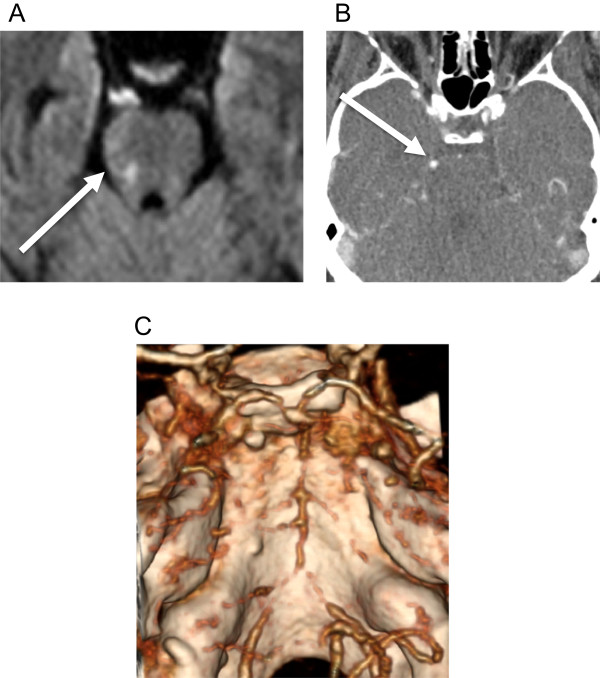
**Right pons ischemia. A**. Diffusion weighted MRI showing restriction in right pons (arrow) consistent with ischemia on October 29, 2012. **B**. Contrast CT with right superior cerebellar artery (arrow) enhancement. **C**. Reformatted 3-D CT angiography on Oct 29, 2012 showing diffuse vasculopathy at the basilar arteries and bilateral vertebral arteries.

**Figure 2:**
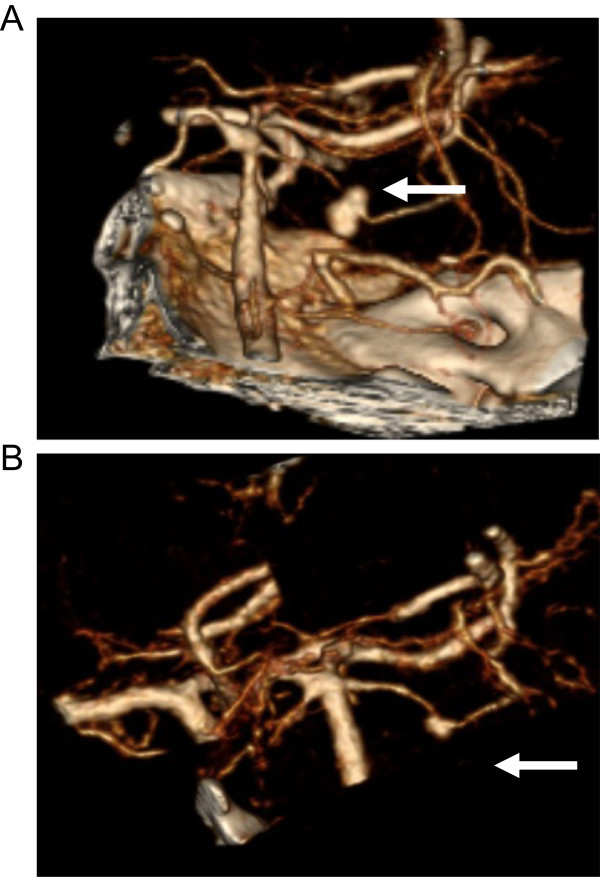
**Superior cerebellar artery aneurysm.** Reformatted 3-D CT angiography of right superior cerebellar artery showing **A**. a 4.2 mm aneurysm (arrow) on Nov 5, 2012 and later **B**. with significant resolution of aneurysm (arrow) after 3 months of amphotericin therapy on Feb 5, 2013.

She presented to this hospital three days after discharge with fevers, worsening headache and vomiting. She also had developed new left face, hand and foot numbness. Neurological exam revealed diminished light touch and temperature sensation in the region of the left cranial trigeminal nerve, mandibular division, left hand and the dorsal aspect of left foot, as well as 4/5 strength on left foot dorsiflexion. Brain magnetic resonance imaging (MRI) on October 21 showed evidence of acute infarct at the right pons with the suggestion of a prominent vessel in the right perimesencephalic cistern suspicious for a mycotic aneurysm and evidence of diffuse vasculopathy of the vertebrobasilar arteries, (Figure 1). A computed tomography (CT) angiography showed a 4.1 mm aneurysm at the right superior cerebellar artery in the right perimesencephalic cistern (Figure 2A). Repeat LP showed stable pleocytosis (Table 1). She was restarted on liposomal amphotericin B (7.5 mg/kg daily) and intravenous voriconazole (6 mg/kg q12h).

One week later, repeat brain MRI with gadolinium showed evidence on gradient echo sequences of subarachnoid signal changes consistent with either blood or inflammation in the prepontine cistern, bilateral occipital horns, and right hemispheric subarachnoid space. The aneurysm appeared stable in size, though more irregular. In consultation with Neurosurgery, the risks of operation given the location, size and friable appearance of the aneurysm outweighed the potential benefits of surgical intervention. She had a MRI lumbar spine due to new onset urinary retention during her hospitalization (November 13^th^) which showed evidence of arachnoiditis affecting intrathecal and bilateral dorsal extradural components. The patient underwent a repeat LP on November 9^th^, now 10 weeks after initial contaminated steroid injection, which indicated persistent pleocytosis (Table 1). The voriconazole level, 5 days after reinitiation of 6 mg/kg intravenous therapy was less than 1 mcg/mL and dosing was increased to 9 mg/kg dosing despite presence of hallucinations on the lower nontherapeutic dose begun at this second hospitalization on October 21^st^.Prior to discharge repeat CT angiography showed slight decrease in aneurysm size to 3.8 mm and no subarachnoid hemorrhage (not shown). Three months into therapy the right superior cerebral artery aneurysm was diminished but not resolved in February, 2013 (Figure 2B).

**Figure 3:**
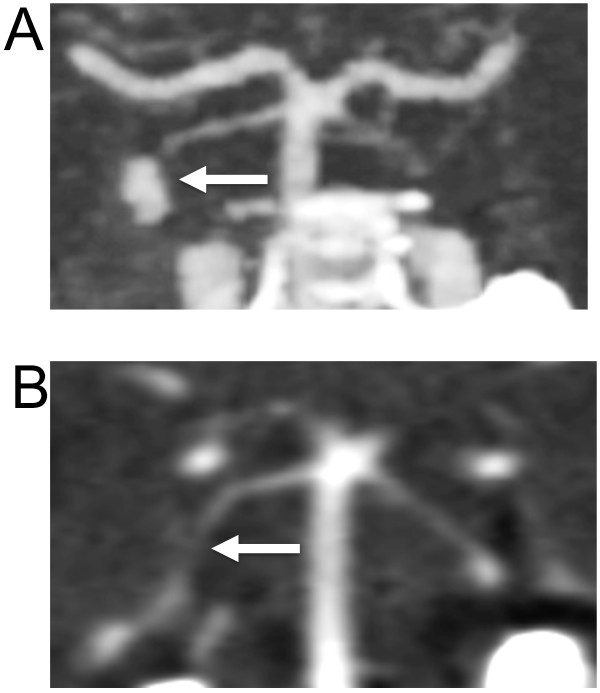
**Resolution of superior cerebellar artery aneurysm.** CT angiography from neck coronal slices in October, 2012 **A**. showing prominent aneurysm (arrow) involving the right superior cerebellar artery with **B**. follow-up on August 2013 indicating resolution of the aneurysm (arrow).

Microbiological studies resulted in negative fungal cerebrospinal fluid (CSF) cultures on October 8, 15, 21 and Novemberz 9 at the hospital clinical laboratory. CSF PCR was also negative for fungal and *Exserohilum species* detection at Centers for Disease Control and Prevention (CDC) [7]. Archival CSF from the three October and single November LPs held in the state lab were retrospectively evaluated for (1,3)-β-D-glucan (BDG), with results showing levels greater than 500 pg/mL (positive >80 pg/mL).She received a total of 32 weeks of antifungal treatment, initially with 14 weeks of combined voriconazole and liposomal amphotericin B, followed by 8 weeks of voriconazole alone due to renal insufficiency from liposomal amphotericin B, followed by 4 weeks of combined therapy and ending with 6 weeks of liposomal amphotericin B alone which was discontinued secondary to renal dysfunction, nausea, emesis and myalgias. She completed 26 weeks of voriconazole and 24 weeks of liposomal amphotericin B. Magnetic resonance angiography (MRA) was performed at the end of antifungal therapy, which showed resolution of the aneurysm. Her symptoms resolved forty weeks after onset. MRA was performed at the end of antifungal therapy, which showed resolution of the aneurysm. An August, 2013 CTA performed 3 months after discontinuation of all antifungal therapy did not show any recurrence of aneurysm (Figure 3). More than a year after onset of symptoms and 6 months off therapy she remains well and is considered a “cure”.

## Conclusions

This case reflects many challenges of diagnosis and treatment of cerebral phaeohyphomycosis. All of her CSF cultures and PCR tests were negative. CSF testing for BDG has not been clinically validated, although BDG levels were elevated in this case [11,12]. BDG is a glucose polymer, a cell wall component of most fungal organisms. The Fungitell assay (Associates of Cape Cod, Inc., Falmouth, MA) has been approved by the US Food and Drug Administration for detection of BDG in human serum and has shown utility in the diagnosis of infections caused by several fungal pathogens [8,9]. Ongoing studies are examining the utility of CSF BDG in CNS fungal infections. Though this patient did not have findings of systemic fungemia, the titers were elevated on multiple occasions, likely reflecting true positive results.

Given the close proximity of the aneurysm to the active inflammation seen on brain MRI, its location distal to a vessel juncture and its poorly defined neck, fusiform and irregular shape, we felt that her aneurysm was mycotic in nature. Patients with unruptured mycotic aneurysms are generally managed with medical therapy alone though there is general consensus that if the mycotic aneurysm enlarges, fails to resolve or ruptures during medical treatment, they should be considered for surgery or endovascular treatment [13]. To our knowledge this is the rare case report of successful medical management of a cerebral mycotic aneurysm related to phaeohyphomycosis in an immunocompetent individual. A recent report detailed three cases of fungal meningitis related to the outbreak that presented with symptoms consistent with ischemic stroke; two cases were found to have mycotic aneurysms on postmortem examination and none had mycotic aneurysm noted on imaging [14]. The two cases unfortunately were diagnosed at autopsy and all three patients had common risk factors for ischemic events; our patient had no traditional risk factors for stroke and had both acute infarct and aneurysm formation noted on imaging with improvement in symptoms and size of aneurysm at one year of follow up.

Voriconazole is known to have erractic pharmacokinetics and despite new onset hallucinations, the 6 mg/kg dosing was not therapeutic emphasizing the importance of mandatory drug levels in all patients on therapy [15]. This patient had a subtherapeutic voriconazole level obtained 5 days after initiation. She was on no medications known to decrease serum levels of voriconazole; this highlights the nonlinear pharmacokinetics of voriconazole and emphasizes the need to monitor levels closely. We can only speculate that the patient, a young, nonobese otherwise healthy adult, metabolized voriconazole at an increased rate. We were able to complete 23 weeks of liposomal amphotericin and 26 weeks of voriconazole with 17 weeks of overlap. While there has been some preclinical data suggesting antagonism between liposomal amphotericin and voriconazole, we elected to treat with dual therapy in accordance with CDC guidance and the severity of her symptoms. Normalization of pleocytosis, erythrocyte sedimentation rate (ESR) and C-reactive protein (CRP) occurred after 12 weeks of therapy. Despite maximum medical therapy, she developed a new 1 cm epidural abscess at the local injection site 4 months into therapy that was sterile by fungal and bacterial culture when surgically removed. Additionally, our patient’s course was complicated by arachnoiditis and adverse drug events (hepatitis, renal insufficiency), despite being otherwise young and healthy.

This case emphasizes the ongoing need for close monitoring of patients exposed during the 2012 fungal meningitis outbreak. Our patient developed meningitis, cerebral mycotic aneurysm, an acute infarct, epidural abscess, arachnoiditis, and stroke symptoms all as a result of the contaminated injection; her course was complicated by adverse drug events necessitating discontinuation of therapy. Complications seen in our case occurred several weeks after initial infection during combination antifungal therapy. Recommendations have evolved over time concerning appropriate duration of therapy, but no guidance exists on serial imaging. This underscores the need to follow patients clinically and radiographically given the difficulties in relying on laboratory evaluation alone. Further studies are needed to determine the utility of CSF BDG in diagnosing and monitoring patients with meningitis thought to be related to fungal infection.

## Consent

Written informed consent was obtained from the patient for publication of this Case Report and any accompanying images. A copy of the written consent is available for review by the Editor-in-Chief of this journal.

## Abbreviations

BDG: (1,3)-β-D-glucan; CDC: Centers for disease control and prevention; CNS: Central nervous system; CRP: C-reactive protein; CT: Computed tomography; CTA: Computed tomography angiogram; ESR: Erythrocyte sedimentation rate; LP: Lumbar puncture; MRA: Magnetic resonance angiography; MRI: Magnetic resonance imaging; PCR: Polymerase chain reaction.

## Competing interests

The authors declare that they have no competing interests.

## Authors' contributions

GN, OF, EF, JB, RL, KJ and DS provided hospital clinical care and SR provided outpatient followup. GN, KT, LW, SR, KJ and DS obtained BDG testing from multiple samples. GN, OF, KT, EF, LW, SR, KJ and DS drafted the manuscript. All authors read and approved the manuscript.
